# Qualitative development of the ‘Questionnaire on Pain caused by Spasticity (QPS),’ a pediatric patient‐reported outcome for spasticity‐related pain in cerebral palsy

**DOI:** 10.1007/s11136-013-0526-2

**Published:** 2013-09-24

**Authors:** Thorin L. Geister, Manjari Quintanar-Solares, Mona Martin, Stephan Aufhammer, Friedrich Asmus

**Affiliations:** 1 Merz Pharmaceuticals GmbH, Eckenheimer Landstraße 100, 60318 Frankfurt am Main, Germany; 2 Health Research Associates, 6505 216th Street SW, Suite 105, Mountlake Terrace, Seattle, WA 98043 USA

**Keywords:** Spasticity-related pain, Cerebral palsy, Patient-reported outcome measure, Children, Content validity, Botulinum toxin

## Abstract

**Purpose:**

To develop a patient-reported outcome measure for spasticity-related pain in children/adolescents (age 2–17 years) with cerebral palsy (CP), the ‘Questionnaire on Pain caused by Spasticity (QPS).’

**Methods:**

Using a semi-structured interview guide, concept elicitation interviews on spasticity-related pain in upper and lower limbs were conducted in 21 children and caregiver pairs. Data were used to modify initial QPS modules and develop six draft modules, which were subsequently refined and finalized in four consecutive cognitive interview waves (12 children and caregiver pairs).

**Results:**

To accommodate the broad range in the children’s communication skills, QPS child/adolescent modules were developed in both interviewer-administered and self-administered formats. With the additional parent modules, three QPS modules were developed for each of the upper and lower limb applications. Information gained from the parent/caregiver modules complements the child/adolescent assessment. Parents report observed signs and frequency of pain in the same situations used to capture the child/adolescent reports of pain severity (e.g., rest, usual daily activities, active mobilization, and physically difficult activities). Participating children/adolescents and parents/caregivers reported that the final QPS instruments were comprehensive, relevant to the child’s spasticity-related experience, and easy to understand and complete.

**Conclusions:**

The QPS is a novel instrument for the assessment of spasticity-related pain in children/adolescents with CP that was developed with direct patient input. Its modules allow the use of this instrument in children/adolescents with varied levels of impairment and communication skills.

## Introduction

Cerebral palsy (CP) is a motor impairment condition caused by damage to the developing brain [1, 2]. Injury to the motor areas may cause spasticity, in approximately 80 % of patients, which is often accompanied by pain. Further, CP-related disorders also include non-motor symptoms such as impaired cognition, behavioral problems, and visual and hearing disorders, which can compromise communication ability. In pediatric patients with spasticity, assessments and therapeutic interventions commonly focus on motor symptoms, but spasticity-related pain (SRP) is often underreported and consequently undertreated [3–7]. SRP can be continuous or recurrent; pain incidence and severity may be affected by movements and different activity situations throughout the day, for example, at rest, when walking, when playing, or during physical therapy [4, 8–10].

Growing evidence suggests that in adults with spasticity, SRP can be decreased by botulinum toxin injections [11, 12]. Even when intramuscular injections of botulinum toxin are currently recommended for pediatric patients with CP [8, 13, 14], most clinical studies conducted in children/adolescents have been small and outcome measures have not been consistent or specifically developed for children with CP.

Assessing pain in children can be challenging, particularly in children with cognitive impairments associated with CP [6]. General measures of pain [15, 16] that are commonly used in children focus on acute or chronic pain [17], or rely on proxy or observational assessment of pain [18]. There are some quality of life (QoL) assessments for children with CP that include a limited number of pain items [19–22]. Tools developed to specifically assess pain in children with CP are tailored for severely disabled children only, where pain behavior is evaluated by healthcare professionals [23] or by the child through scoring of daily situation drawings [24]. To date, there are no instruments available that specifically assess SRP in children with CP for use in clinical trials of botulinum toxin and other therapies. Therefore, the therapeutic benefit of botulinum toxin for SRP in children with CP needs to be studied using well-developed outcome measures. Here, we report the qualitative development and documentation of content validity for the ‘Questionnaire on Pain caused by Spasticity’ (QPS), a patient-reported outcome (PRO) and observer-reported outcome (ObsRO) for the assessment of SRP in children with CP.

## Methods

### Study design

**Fig. 1:**
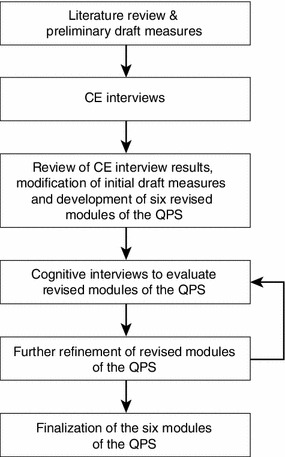
Chronology of activities for developing the QPS. *CE* concept elicitation, *QPS* Questionnaire on Pain caused by Spasticity

The chronology of activities in the development of the QPS is summarized in Fig. 1 and comprised four principal phases: (1) literature review, (2) concept elicitation (CE) interviews, (3) QPS revision, and (4) field testing with cognitive interviews. The development of the QPS conformed to the United States Food and Drug Administration (FDA) and other good research practice guidelines, which point out the importance of establishing proper content validity for PRO’s and ObsRO’s in the target patient population [25–27]. This study adhered to ethical principles for human research studies and to good clinical practice guidelines [28]. Appropriate institutional review board approval was obtained before study activities began. Caregivers received financial compensation for their time and travel expenses associated with the study participation.

### Literature review and preliminary draft measures

The decision to start with the QPS development was based on a qualitative publications search in the PubMed database (search terms: spasticity, pain, cerebral palsy, children, and botulinum). The aim was to identify the current peer-reviewed literature on SRP and available pain assessments in children with CP and for botulinum toxin treatment. General information about SRP was reviewed, and findings on important concepts were outlined in four initial draft questionnaire modules.

### Concept elicitation interviews

CE interviews were conducted with the child/adolescent and their parent/caregiver, following a semi-structured interview guide. Initial open-ended questions explored the presence of spasticity and SRP in patient language (e.g., ‘What happens when your (leg/arm) gets tight or stiff?’ ‘Does your (leg/arm) hurt sometimes?’ and ‘What happens when it hurts?’). These questions were followed by probing questions that explored the presence of the latter symptoms in particular situations (e.g., ‘Do your arms ever tighten up when you’re doing things like getting washed and dressed?’). Interviewers also recorded visual clues, such as children’s facial expressions and pointing, and explored whether the Wong-Baker FACES^®^ scale [29] was understood by the children and acceptable to the adolescents.

Interviews were audio-recorded, transcribed, and coded using Atlas.ti software for content analysis. Inter-rater agreement between coders was assessed on approximately 10 % of the transcript database. To assess saturation of concepts, transcripts were ordered chronologically, grouped into quartiles, and the newly appearing concept codes for each new transcript group were compared to the prior group. Saturation of concept was determined to have been reached when there were no longer new concepts being coded, and thus, all relevant concepts were captured. Based on previous experience conducting qualitative research and the degree of homogeneity in this population, we estimated that saturation of concept would likely be achieved within 14–16 CE interviews.

The study was conducted at four different clinical sites in the USA with three trained and experienced interviewers. Cognitive interviews were subsequently conducted at two of the four sites. Sites were selected in different geographic areas for their large pool of eligible subjects, to avoid the clustering of regional effects. Sites were specialized for treatment of children with CP and were regularly administering therapies such as botulinum toxin, baclofen, or phenol to minimize spasticity. Patient records were initially reviewed by clinical staff to identify suitable families, and primary caregivers were approached if eligible.

Purposive sampling was used to maximize variation in both CE and cognitive interviews, so children in different age brackets and with different gross motor function classification system (GMFCS) levels were recruited. Male and female children (2–11 years) and adolescents (12–17 years) with unilateral or bilateral CP with spasticity and intermittent SRP in either the upper limb or the lower limb (or both) on at least a weekly basis were eligible for participation. Children were categorized into the following age bands for recruitment: 2–4, 5–7, and 8–11-year old. To include the full spectrum of children with CP, recruitment targeted children with upper or lower limb SRP and with GMFCS of I–III (ambulatory, less impaired) and IV–V (non-ambulatory, greater impairment). Subjects had already received intramuscular botulinum toxin treatment of spasticity or were likely candidates for this type of treatment. Subjects were excluded if they had fixed contractures, predominant forms of muscle hypertonia other than spasticity, constant pain, pure, or predominantly dyskinetic CP, or had undergone surgery for pes equinus in the 12 months prior to recruitment. Subjects unable to answer interview questions due to profound cognitive impairment were also excluded.

### QPS revision

The CE interview results guided revisions of the initial draft measures to develop the preliminary modules of the QPS. Content validity assessment included the relevance of concepts to subjects and caregivers, the specific language used to describe symptoms and observed pain behaviors, the appropriateness of the aspects of each concept being measured (frequency, severity, duration of pain), and the appropriateness of the recall period.

### Cognitive interviews

In order to field test the QPS, four separate waves of cognitive interviews were conducted (approximately four interviews per wave) during which subjects and their caregivers were asked to complete a preliminary module of the QPS. Participants were asked to ‘think aloud’ and describe their thought process for interpreting each question and arriving at an answer. A sample of the questions asked would be ‘Please tell me in your own words what this item is asking you about’ (for caregivers) or ‘What did you remember when you read this question?’ (for children).

The cognitive interview process was used to assess and document the participant’s understanding of the underlying concept presented in the QPS items and to refine language and restructure difficult items for children and caregivers. Other aspects of the preliminary QPS were also assessed, including the appropriateness of the recall period, the fit of the response options, the suitability of the Wong-Baker FACES^®^ scale [29], and overall clarity of the format and instructions.

An additional 12 child and parent/caregiver pairs were enrolled in four separate waves of the cognitive interviews to assess the preliminary measure.

## Results

### Literature review and preliminary draft measures

The main findings of the literature review can be summarized as followed: (1) Pain in children with CP is an under-recognized and emerging topic in this population due to the significant influences on QoL [3, 7–9]; (2) SRP is one of multiple pain sources and needs to be distinguished from others such as hip dislocation, dystonia, surgeries, or contractures [6, 10]; (3) several general, acute pain assessments, and QoL questionnaires with pain items are available, but none specific for SRP in children and adolescents with CP and following actual guidelines [15, 16, 25–27]; (4) pain severity, frequency, and location in dependence on different activity situations are important [8, 10, 16]; and (5) a modular approach for an assessment is necessary to differentiate between upper and lower limb SRP and to account for different ages or cognitive abilities of the patients [1, 8]. This supports the development of a parent/caregiver module to capture observed signs of SRP in children who were either too young or too impaired to communicate. Based on the review, four initial draft modules were created: one upper and lower extremity child/adolescent module and one upper and lower extremity parent/caregiver module. These modules corresponded to each other in structure and item content across a general pain item and three different activity situations (at rest, usual activities, and active mobilization) plus an item on location of pain. A 7-day recall period was selected based on considerations that (1) SRP is characterized as intermittent and not constant pain, so sufficient time was needed for the subject to have experienced pain to report about; (2) specific activities such as physical exercises and clinical therapy sessions relate more to a weekly schedule than a daily one; and (3) qualitative evidence with both children and parents indicated reliable memory for the past week (7 days) for activity and pain reporting, but not beyond that.

### Concept elicitation interviews

CE interviews were conducted with 21 children (aged 2–16 years) and caregiver pairs (Table 1 ). Subjects’ cognitive skills varied widely within age groups and GMFCS levels. Findings confirmed that SRP and spasticity itself were relevant to children and caregivers and affected their everyday lives. In total, 40 relevant symptom concepts were identified in the CE interviews. Larger domains such as ‘pain related to spasticity’ included sub-concepts such as ‘pain at rest,’ ‘pain triggered by specific activities,’ ‘pain severity,’ or ‘pain frequency’.

**Table 1:** Child and caregiver population and demographics

	Concept elicitation interviews	Cognitive interviews
Children/adolescents	(*n* = 21)	(*n* = 10^a^)
Age (years)		
Mean (SD)	9.5 (3.8)	12.1 (2.8)
Range	2–16	7–16
Female, *n* (%)	6 (28.6)	5 (50.0)
Ethnicity, *n* (%)		
White (non-Hispanic)	16 (76.2)	5 (50.0)
White (Hispanic)	4 (19.0)	3 (30.0)
Hispanic/Latino	0	2 (20.0)
Black/African American	1 (4.8)	0
Cognitive impairment for age (reported by caregivers), *n* (%)	7 (33.3)	3 (30.0)
GMFCS evaluation		
GMFCS Level I–III	12 (57 %)	5 (50 %)
GMFCS Level IV–V	9 (43 %)	5 (50 %)
Localization of pain, *n* (%)		
Both lower limbs only	2 (9.5)	3 (30.0)
One lower limb only	1 (4.8)	0
One upper and one lower limb, same side	6 (28.6)	0
Upper and lower limb(s) on both sides	12 (57.1)	7 (70.0)
Caregivers	(*n* = 21)	(*n* = 11^a^)
Age (years)		
Mean (SD)	36.1 (7.7)	42.0 (7.3)
Range	22–58	32–53
Female, *n* (%)	20 (95.2)	10 (90.9)

*GMFCS* Gross Motor Function Classification System, *SD* standard deviation

^a^Twelve pairs (10 children and 11 caregivers) were enrolled for the cognitive interviews. Two caregivers had children who were too young to be interviewed, and one caregiver was the mother of a pair of participating twins (and was interviewed twice)

Saturation of concept was achieved by the fourth transcript group. Inter-rater agreement was between 92.3 and 94.9 % for the assignment of specific concept codes.

The most frequently mentioned symptom-related concept was ‘Pain experienced in general,’ with 50 expressions (10.9 % of all symptom expressions) in the child interviews and 36 expressions (7.8 % of all symptom expressions) in the caregiver interviews. This code category mainly captured children saying ‘it hurts,’ and caregivers reporting ‘pain’ and ‘hurt.’ The pain experienced by the children differed depending on the type of activity they engaged in. The next most frequently expressed symptom concept was ‘tightness’ (used to describe spasticity), with 27 expressions in child interviews (5.9 %) and 62 in caregiver interviews (13.5 %).

Generally, the children were not able to describe frequency or duration of their SRP in a reliable way, but they could identify pain intensity/severity using the Wong-Baker FACES^®^ scale [29].

All caregivers reported having observed SRP behaviors in their children during the last 7 days; yet, only 16 (76.2 %) of the children acknowledged having pain. One child directly stated he would deny pain: ‘Sometimes it hurts so bad, but I’ll never tell you.’ Caregivers most frequently detected pain by ‘observing body movement’ (*n* = 113, 17.4 % of the caregivers’ expressions for pain detection) followed by other signs such as ‘the child articulating pain,’ ‘changing the position of their body,’ ‘having mood changes,’ and ‘having different facial expressions’ [*n* = 102 (15.7 %); *n* = 68 (10.5 %); *n* = 45 (6.9 %); and *n* = 39 (6.0 %), respectively]. Most caregivers were able to report on the frequency of pain based on their observations of pain behaviors and their child’s verbalization of pain.

### QPS revision

CE interviews confirmed that certain children were able to communicate about their pain, but did not have the cognitive or motor abilities to answer a questionnaire on their own. Accordingly, interviewer-administered modules were needed to be developed to cover a higher percentage of the target population. A total of six QPS modules were next drafted (Table 2): two child/adolescent self-administered modules, two child/adolescent interviewer-administered modules, and two parent/caregiver modules.

**Table 2:** The final six modules of the QPS

Upper extremity assessment	Lower extremity assessment
Parent/caregiver observational report module	Parent/caregiver observational report module
Child/adolescent self-administered module	Child/adolescent self-administered module
Child/adolescent interviewer-administered module	Child/adolescent interviewer-administered module

Content analysis of the CE interview results determined the selection of 11 symptom concepts (assessed in 11 of 12 items) for the child/adolescent modules and 12 symptom concepts (assessed in 12 of 17 items) for the parent/caregiver modules of the QPS (Table 3). It was also confirmed that a 7-day recall period was appropriate for this age group.

**Table 3:** Concepts in spasticity-related pain selected for inclusion in the QPS

Child/adolescent modules (upper and lower extremity)			Parent/caregiver modules (upper and lower extremity)		
Targeted symptom concepts	Item number	Response scale	Targeted symptom concepts	Item number	Response scale
Spasticity	1	Yes/no	Spasticity (observed)	5	Yes/no
General spasticity-related pain (SRP)	2	Yes/no	General SRP (verbalization)	6	Yes/no
General SRP severity	3	WBF^a^	General SRP (observed signs)	7	Yes/no
SRP while at rest	4	Yes/no	General SRP observed frequency	8	Frequency^b^
SRP while at rest severity	5	WBF^a^	SRP while at rest (observed signs)	9	Yes/no
SRP during usual activities	6	Yes/no	SRP while at rest observed frequency	9b	Frequency^b^
SRP during usual activities severity	7	WBF^a^	SRP during usual activities (observed signs)	10	Yes/no
SRP during active mobilization	8	Yes/no	SRP during usual activities observed frequency	10b	Frequency^b^
SRP during active mobilization severity	9	WBF^a^	SRP during active mobilization (observed signs)	11	Yes/no
SRP during difficult activity	11	Yes/no	SRP during active mobilization observed frequency	11b	Frequency^b^
SRP during difficult activity severity	12	WBF^a^	SRP during difficult activity (observed signs)	13	Yes/no
			SRP during difficult activity observed frequency	13b	Frequency^b^

Items not described in this table collect additional information of interest such as role of the caregiver, definitions, pain location, etc.

*QPS* Questionnaire on Pain caused by Spasticity, *SRP* spasticity-related pain

^a^WBF: Wong-Baker FACES^®^ scale, includes six faces ranging from smiling to crying with scores of *no hurt* (0), *hurts little bit* (2), *hurts little more* (4), *hurts even more* (6), *hurts whole lot* (8), and *hurts worst* (10)

^b^The frequency scale includes five points with scores of *never* (0), *rarely* (1), *sometimes* (2), *often* (3), and *always* (4)

**Fig. 2:**
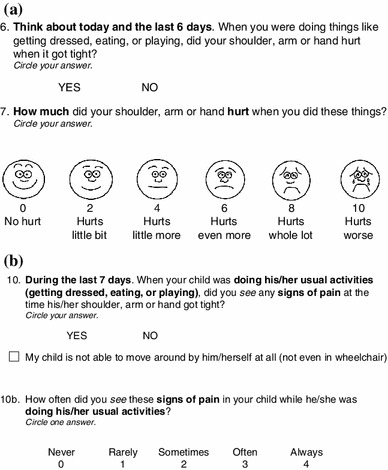
Item examples of the final QPS. **a** Item 6 and 7 of the upper extremity child/adolescent module. **b** Item 10 of the upper extremity parent/caregiver module

Items in the four child/adolescent modules probe the occurrence of SRP (‘Yes/No’) and pain intensity (Wong-Baker FACES^®^ scale) in different activity situations (pain in general, at rest, during usual activities, during physical therapy, and while performing a self-defined very difficult task) during the last 7 days. The caregiver modules probe for behaviors caregivers recognize as indicating pain and how frequently these behaviors were observed in the different activity situations on a five-point response scale from 0 = never to 4 = always. An example item of the upper extremity child/adolescent module and the corresponding items in the parent/caregiver module are presented in Fig. 2. Pain location was evaluated by caregivers and children together and included only in the caregiver’s questionnaire.

### Cognitive interviews

Cognitive interviews were conducted with 12 child (aged 7–16 years) and caregiver pairs. The demographic and clinical characteristics of subjects and caregivers are detailed in Table 1.

#### Cognitive interviews with children and adolescents

The formatting and wording of the questionnaire were amended in response to difficulties identified during the interview waves. For instance, the phrase ‘think about the last week’ made younger children think about the pain events in the week prior to the interview, rather than the week of the interview. Therefore, the wording was first amended to ‘in the last week’ and then to ‘think about today and the last 6 days’ when it became apparent that adolescents were not considering ‘today’ when asked about pain events ‘in the last week.’ In the fourth wave of cognitive interviews, all subjects interviewed considered the full range of the last 7 days for their answers, indicating that the wording of the recall period was adequate. Some children with CP denied their pain, similar to observations in the CE interviews.

#### Cognitive interviews with parents and caregivers

Most changes to the wording of the QPS were made after the first interview wave. Examples include the addition of ‘tightness’ as a descriptor next to ‘spasticity’ throughout the QPS, making a clearer distinction between nonverbal signs of pain and verbal expressions. Some caregivers selected their answers thinking of the last time they had observed signs of pain in a given activity situation, which in some cases went beyond the desired 7-day recall period. Therefore, clinic staff administering the QPS will be trained to verbally remind of the recall period. Table 4 illustrates changes made to the parent/caregiver QPS during the cognitive interview waves for an example item (SRP at rest). At the completion of the fourth wave of cognitive interviews, no further wording or format changes were needed for either the child/adolescent or parent/caregiver modules of the QPS.

**Table 4:** Example of the refinement of the QPS in cognitive interviews

Items evaluated in Wave 1	Items evaluated in Wave 2	Items evaluated in Wave 3	Items evaluated in Wave 4
Instructions: Now, we will ask you about your child’s pain caused by spasticity in some specific situations	Instructions: Now, we will ask you about your child’s pain caused by spasticity (**tightness**) in some specific situations	Instructions: Now, we will ask you about your child’s pain caused by spasticity (tightness) in some specific situations	Instructions: Now, we will ask you about your child’s pain caused by spasticity (tightness) in some specific situations
Item 9—lower During the last 7 days, when your child was at rest (sitting, relaxing, watching TV, sleeping), did you see signs of pain at the time his/her hip, leg, or foot got tight? Circle your answer	Item 9—lower During the last 7 days, when your child was at rest (sitting, relaxing, watching TV, sleeping), did you see signs of pain at the time his/her hip, leg, or foot got tight? Circle your answer	Item 9—lower During the last 7 days, when your child was at rest (sitting, relaxing, watching TV, sleeping), did you see **any** signs of pain at the time his/her hip, leg, or foot got tight? Circle **one** answer	Item 9—lower During the last 7 days, when your child was at rest (**relaxing, watching TV, sleeping**), did you see any signs of pain at the time his/her hip, leg, or foot got tight? Circle one answer
Item 9—upper During the last 7 days, when your child was at rest (sitting, relaxing, watching TV, sleeping), did you see signs of pain at the time his/her shoulder, arm, or hand got tight? Circle your answer	Item 9—upper During the last 7 days, when your child was at rest (sitting, relaxing, watching TV, sleeping), did you see signs of pain at the time his/her shoulder, arm, or hand got tight? Circle your answer	Item 9—upper During the last 7 days, when your child was at rest (sitting, relaxing, watching TV, sleeping), did you see **any** signs of pain at the time his/her shoulder, arm, or hand got tight? Circle **one** answer	Item 9—upper During the last 7 days, when your child was at rest (**relaxing, watching TV, sleeping**), did you see any signs of pain at the time his/her shoulder, arm, or hand got tight? Circle one answer
Yes/no	Yes/no	Yes/no	Yes/no

This table illustrates typical changes that were made to the draft measures over the four waves of cognitive interviews, using an example item from the caregiver module of the QPS. Changes are italicized

*QPS* Questionnaire on Pain caused by Spasticity

During the cognitive interviews, children and caregivers judged the QPS to be easy to understand, complete, and relevant to their experience with SRP. A reference manual was developed to help guide clinical staff in selecting the appropriate module of the child/adolescent QPS (self-administered vs. interviewer-administered), based on the subjects’ motor and cognitive skills, and to help with any problems that might occur.

## Discussion

The QPS was developed as a novel PRO and ObsRO instrument for assessing SRP in children and adolescents with CP from 2 to 17 years. Qualitative results from the CE interviews and from the cognitive interviews supported concept relevance for both children and their parents.

The most common pain assessment instruments used in very young children are proxy reported, such as the Children’s Hospital of Eastern Ontario Pain Scale (CHEOPS) [30] or the Faces, Legs, Activity, Cry, and Consolability (FLACC) Pain Assessment Tool [31]. The widely used OUCHER!™ scale has been developed to assist 3- to 12-year olds to describe pain intensity [32]. All of these tools evaluate acute pain, for example, after surgery. The QPS is specifically designed to assess chronic SRP in children with CP aged 2 years and older.

The review of the available literature highlighted that SRP can be continuous or recurrent and varies in the type of trigger, intensity, frequency, or duration according to different activity situations [8]. CE interviews ensured that the QPS allows for complete evaluation of SRP by assessing pain triggered by different key activity situations. In our study, most children could only reliably report pain severity, but not pain frequency or duration. Therefore, the child/adolescent modules of the QPS only ask about pain intensity. The parent/caregiver can only report observed signs of pain and the frequency of those signs; consequently, the parent/caregiver modules of QPS only report on observed signs of pain and their frequency, but not on the subjective experience of pain intensity. Since the qualitative results showed that pain duration was not reliably reported by either the children or their parents/caregivers, it is not addressed in any module of the QPS.

Qualitative results from both CE and cognitive interviews helped to ensure that the QPS used language appropriate for assessing pain in younger children. Most children and many of the parents/caregivers could not relate to the term ‘spasticity.’ The children reported experiencing ‘tightness,’ and therefore, this was the term used in the QPS modules for both children and parents. The Wong-Baker FACES^®^ scale has previously been shown to be easy to use and preferred by children, parents, and healthcare professionals when compared with other faces pain scales [33]. We found the scale to be both appropriate and helpful for this population of children with CP, and it was therefore incorporated into the QPS.

A suitable recall period should provide a more integrated assessment than a description of pain at a particular moment [34]. However, a recall period that is too long may be difficult to remember accurately, especially for younger or intellectually impaired children [35]. Previous studies have reported the successful use of a 1-week recall period with children [36, 37]. During the cognitive interviews, parents and children could clearly relate events to the 7-day timeframe, confirming that this recall period was appropriate for the QPS.

The six-specific modules of the QPS are a novel feature of this instrument. This unique design allows information on symptom severity to be obtained from very young or cognitively impaired children through the use of an interviewer-administered module, without the loss of important data due to age, insufficient reading skills, or communication impairment. Some children with CP will be capable of self-administering the QPS, while others lacking sufficient motor or reading skills will be able to complete the interviewer-administered module. Additionally, the parent/caregiver module of the QPS allows parents or caregivers to provide valuable information on behalf of children who are too young or unable to communicate due to cognitive impairments. Each of the three modules has versions for upper and lower extremities for the separate assessment of spasticity in these parts of the body.

Interestingly, some of the children interviewed were reluctant to admit having pain; some would not even admit having spasticity. This qualitative study confirmed that this was a conscious denial of SRP and not due to the child’s inability to recognize pain or to understand the concept of spasticity as ‘tightness.’ However, as shown in the cognitive interview process, the unique design and flow of the QPS was able to depict children’s initial denial of pain with an early general question and then subsequently capture the admission of pain in the more detailed questions regarding different activity situations that trigger pain.

One critical point of a qualitative study is appropriate sample size. Sample size is hard to define for a PRO, but should be guided by the heterogeneity of the population and the complexity of concepts. Saturation of concept is then the key criterion to determine appropriateness of the chosen sample size [26], which was successfully demonstrated in this study. The predominant factor influencing the heterogeneity in the population is the varying clinical presentation of CP with respect to motor skills (upper and lower limb, unilateral vs. bilateral), cognition (about 50 % of children show relevant impairment), learning, as well as hearing and seeing [1]. Using a pragmatic approach, we concluded that CP subjects with marked limitation of cognition and communication would not be able to contribute to the initial phases of the development of a measure for SRP. We therefore aimed to have as much input from CP children and adolescents who were able to communicate. Due to the implementation of broad input from pediatric subjects, we succeeded in building a measure that is clear and has simple concepts. Vocabulary was aimed to fit the younger children and yet still be acceptable to the older ones. Since a 12-year-old child with CP may have the communication skills of a 5-year old, the best approach was to tailor the measures to the lowest reading level and to suggest a decision-making process for selecting which version was most appropriate for a given child. Hence, practical decision criteria such as motor skills, cognition, and the ability to read and write are important for a PRO measurement strategy in this population than calendar age categories. Given the demonstrated saturation of concept in the CE interviews and the positive feedback in the cognitive interviews, there is good evidence that the chosen methodology and sample sizes adequately support content validity of the QPS in this population.

Ease of use and appropriate understanding of concept are supported by the qualitative work and further supported by the initial translation and linguistic validation process that is currently under way to establish QPS translations for other countries. The next step in the development of the QPS is a psychometric validation study. This study and the clinical studies to follow will cover an even greater diversity in the target population including a large age range of children with different reading levels as well as different sensory, motor, and cognitive impairments. In addition to performance characteristics such as reliability and validity, the ability of the QPS to detect and quantify improvements in SRP in clinical trials of botulinum toxin treatment for spasticity in children with CP still needs to be verified.

In summary, the QPS is the first PRO and ObsRO instrument specifically developed for the assessment of SRP in children and adolescents with CP. Importantly, the QPS takes into account the special features of this patient population, such as motor, cognitive, and communication impairments that have not been addressed in other pediatric PRO instruments. The QPS aims to allow clinicians and researchers to reliably measure SRP in a way that is meaningful to the children and adolescents with CP as well as their parents or caregivers. This PRO and ObsRO has been developed to monitor the effect of therapeutic interventions such as botulinum toxin injections on SRP to help optimize treatment outcomes.

The QPS can be obtained by emailing the corresponding author (scales@merz.de) at Merz Pharmaceuticals GmbH.

## Abbreviations

CE: Concept elicitation
CHEOPS: Children’s Hospital of Eastern Ontario Pain Scale
CP: Cerebral palsy
FDA: Food and Drug Administration
FLACC: Faces, Legs, Activity, Cry, and Consolability
GMFCS: Gross Motor Function Classification System
ObsRO: Observer-reported outcome
PRO: Patient-reported outcome
QoL: Quality of life
QPS: Questionnaire on Pain caused by Spasticity
SRP: Spasticity-related pain
